# Sequential Waves of Gene Expression in Patients with Clinically Defined Dengue Illnesses Reveal Subtle Disease Phases and Predict Disease Severity

**DOI:** 10.1371/journal.pntd.0002298

**Published:** 2013-07-11

**Authors:** Peifang Sun, Josefina García, Guillermo Comach, Maryanne T. Vahey, Zhining Wang, Brett M. Forshey, Amy C. Morrison, Gloria Sierra, Isabel Bazan, Claudio Rocha, Stalin Vilcarromero, Patrick J. Blair, Thomas W. Scott, Daria E. Camacho, Christian F. Ockenhouse, Eric S. Halsey, Tadeusz J. Kochel

**Affiliations:** 1 Naval Medical Research Center, Silver Spring, Maryland, United States of America; 2 Henry M. Jackson Foundation for the Advancement of Military Medicine, Rockville, Maryland, United States of America; 3 US Naval Medical Research Unit No. 6, Lima, Peru; 4 Laboratorio Regional de Diagnóstico e Investigación del Dengue y otras Enfermedades Virales (LARDIDEV), Instituto de Investigaciones Biomédicas de la Universidad de Carabobo (BIOMED-UC), Maracay, Venezuela; 5 Division of Retrovirology, Walter Reed Army Institute of Research, Silver Spring, Maryland, United States of America; 6 Department of Entomology, University of California at Davis, Davis, California, United States of America; 7 US Naval Medical Research Unit No. 2 – Pacific, American Embassy Singapore, Singapore; 8 Division of Malaria Vaccine Development, Walter Reed Army Institute of Research, Silver Spring, Maryland, United States of America; Oxford University Clinical Research Unit, Vietnam

## Abstract

**Background:**

Dengue virus (DENV) infection can range in severity from mild dengue fever (DF) to severe dengue hemorrhagic fever (DHF) or dengue shock syndrome (DSS). Changes in host gene expression, temporally through the progression of DENV infection, especially during the early days, remains poorly characterized. Early diagnostic markers for DHF are also lacking.

**Methodology/Principal Findings:**

In this study, we investigated host gene expression in a cohort of DENV-infected subjects clinically diagnosed as DF (n = 51) and DHF (n = 13) from Maracay, Venezuela. Blood specimens were collected daily from these subjects from enrollment to early defervescence and at one convalescent time-point. Using convalescent expression levels as baseline, two distinct groups of genes were identified: the “early” group, which included genes associated with innate immunity, type I interferon, cytokine-mediated signaling, chemotaxis, and complement activity peaked at day 0–1 and declined on day 3–4; the second “late” group, comprised of genes associated with cell cycle, emerged from day 4 and peaked at day 5–6. The up-regulation of innate immune response genes coincided with the down-regulation of genes associated with viral replication during day 0–3. Furthermore, DHF patients had lower expression of genes associated with antigen processing and presentation, MHC class II receptor, NK and T cell activities, compared to that of DF patients. These results suggested that the innate and adaptive immunity during the early days of the disease are vital in suppressing DENV replication and in affecting outcome of disease severity. Gene signatures of DHF were identified as early as day 1.

**Conclusions/Significance:**

Our study reveals a broad and dynamic picture of host responses in DENV infected subjects. Host response to DENV infection can now be understood as two distinct phases with unique transcriptional markers. The DHF signatures identified during day 1–3 may have applications in developing early molecular diagnostics for DHF.

## Author Summary

The clinical outcome of DENV infection in humans can be DF or the more severe DHF and DSS. The individual's previous DENV exposure history, infecting serotypes, and host genetics are thought to be contributing factors to dengue disease severity. Our study contributed to the current dengue research field in the following ways: 1) Our study reveals the dynamics of host gene expression over each day post onset of symptoms. The gene transcription patterns enabled classification of dengue disease into 2 subtle phases: early acute and late acute. 2) The study identified gene markers differentiating severe dengue cases from non-severe cases with >90% accuracy. Taken together, our study offers insight into host responses in DENV-infected subjects and these results may be valuable for the future development of diagnostic tools for disease severity.

## Introduction

Dengue viruses (DENV) are arthropod-borne single stranded RNA viruses of the family Flaviviridae, genus *Flavivirus*. There are 4 closely related, but antigenically distinct serotypes, DENV-1,-2,-3, and -4. DENVs are endemic in more than 100 tropical and subtropical countries of the world. Presently, no specific therapies or vaccines are available to treat the diseases or to prevent DENV transmission [1], [2].

Typically, febrile illnesses begin 5–7 days from the initial DENV infection by an infected mosquito. The virus propagates in the human host and the viremia titer in the host's peripheral blood peaks during the early days (first 2–3 days) of acute illness, which is then followed by a steep decline [3]. Illnesses caused by DENV infection include undifferentiated dengue fever (DF), dengue hemorrhagic fever (DHF), and dengue shock syndrome (DSS) [4], [5]. According to the WHO's 1997 guidelines [6], [7], DF is an acute febrile illness with two or more manifestations of headache, retro-orbital pain, myalgia, arthralgia, rashes, etc. Symptoms of DF can last for 2–7 days. A DHF case is diagnosed by persistent high fever, hemorrhage tendency, signs of plasma leakage (≥20% increase in hemoconcentration or hypoprotemia, pleural effusion or ascites), and thrombocytopenia (platelet counts ≤100,000/mm^3^). DHF is further classified into 4 grades according to the severity of bleeding and plasma leakage. DSS refers to DHF grades III and IV, which, if not diagnosed and treated in a timely manner, can lead to death [6]–[9].

The pathogenic feature of severe dengue disease is a transient increase in vesicular permeability, resulting in plasma leakage. DENV-infection rigorously activates the host innate immune system, which contributes to early anti-viral defense, but may concomitantly also contribute to the development of plasma leakage [3], [10]. Analyses of host genome-transcript patterns have revealed multiple gene expression profiles associated with dengue infection: NF-κB-initiated immune responses [11], type I interferon (IFN), ubiquitin proteasome pathway [11], cell cycle and endoplasmic reticulum (ER) [12], genes involved in T and B cell activation, surface marker expression, immunoglobulin, complement activation, and inmate immunity [12]–[14]. Some of these genes are differentially expressed in DF vs. DHF or DSS patients [12]–[14]. These studies demonstrated a powerful way for exploring the roles of host immune responses to DENV infection, as well as identifying disease severity markers using peripheral blood mononuclear cells (PBMCs) without purifying the immune cell subsets or manipulating the cells.

In this study, the dynamics of gene expression were analyzed in cohorts of clinically defined DF and DHF subjects. The study established an evolving picture of the host immune response throughout the entire progression of dengue disease, from as early as fever onset to early defervescence. In addition, the study identified key genomic signatures that might be useful for the future development of early diagnosis of severe dengue disease.

## Materials and Methods

### Ethics

All of the procedures were conducted in accordance with the ethical standards of the Naval Medical Research Center Institutional Review Board and with the Helsinki Declaration of 1975, as revised in 1983. Prior to participating in the study, an informed written consent was obtained from each participant or from their parents or legal guardians. The Naval Medical Research Center Institutional Review Board, in compliance with all Federal regulations governing the protection of human subjects, approved the study protocol (NMRCD.2005.0007). This study was also approved by the Comité de Bioética del Instituto de Investigaciones Biomédicas de la Universidad de Carabobo (CBIIB-UC), in Maracay, Venezuela.

### Patient enrollment, diagnosis of DENV, clinical monitoring and specimen collection

Approximately 2500 residents in the city of Maracay, Venezuela, participated in an active surveillance program for DENV transmission. The subjects were monitored for febrile illness through visits or phone calls three times a week. The subjects who were five years of age and older, with a fever duration of less than 120 hours and with two or more of the following symptoms: myalgia, athralgia, leucopenia, rash, headache, lymphadenopathy, nausea, vomiting, positive tourniquet test, thrombocytopenia and hepatomegaly (World Health Organization, Dengue hemorrhagic fever: diagnosis, treatment, prevention and control. 2nd ed. Geneva: WHO; 1997), were enrolled in this study by nurses and physicians of the field team from the Febrile Surveillance Cohort, and by site physicians from local outpatient clinics (Ambulatorio 23 de Enero, Ambulatorio la Candelaria, Ambulatorio Efrain Abad Y Ambulatorio del Norte), and from two metropolitan reference hospitals (Hospital Central de Maracay and Hospital Ivss Jose Maria Carabaño Tosta). Blood samples were obtained by standard venipuncture procedures in a Vacutainer® collection tube with anticoagulant. An initial blood sample was taken upon enrollment for DENV serotype determination using Taqman-based RT-PCR. Only if DENV infection was confirmed by RT-PCR, then serial blood samples were collected at 24, 48 and 72 hours following the initial sample, and one to two samples within 0–72 hours post-fever defervescence and one sample at ≥20 days (convalescent period) following the initial sample. Viremia levels were measured using quantitative RT-PCR at enrollment and at 24, 48 and 72-hour specimens. Clinical symptoms were monitored and recorded at every visit. Separation of plasma and PBMCs was performed by gradient centrifugation over Histopaque-Ficoll (Sigma, St. Louis, MO). The plasma and PBMCs were stored at −70°C.

For the confirmed DENV cases, serum IgM was measured from the samples at acute phase and convalescent phase using enzyme-linked immunosorbant assays (ELISA) [15], [16]. The primary infection was determined using IgM serology: elevated IgM titers (≥1∶100) without detectable PRNT in the acute sample, and elevated IgM levels in the convalescent sample. To determine the prevalence and cumulative incidences of DENV infections, PRNT titers were measured in the sera obtained from acute specimens [17] as previously described [16], [18].

### Hematological analysis

Complete blood cell counts were performed for each blood sample collected using the QBC automated system according to the manufacturer's instructions (Becton-Dickinson 1996). The QBC STAR measures 9 important CBC hematological parameters: hematocrit, hemoglobin, MCHC (mean corpuscular hemoglobin concentration), platelet count, white blood cell count, granulocyte count and percentage, and lymphocyte/monocyte count and percentage.

### RT-PCR

Viral RNA was prepared from 140 µL sera using QIAamp ViralRNA Mini Kits according to the manufacturer's instructions (Qiagen Inc., Valencia, CA, USA). Briefly, the TaqMan® One-Step RT-PCR Master Mix Reagents (PN:4309169, Applied Biosystems) were prepared in the following manner: final concentration of primers, 1 µM; probes, 0.22 µM in a final reaction volume of 25 µl [19]. Thermocycling was set to 50°C for 30 min and 95°C for 10 min, followed by 45 cycles of 95°C for 15 sec and 60°C for 1 min. The PCR reactions were performed in a 7500 Real-Time PCR System (Applied Biosystems). DENV serotype-specific RT TaqMan PCR was performed on the acute samples using a protocol previously described by Laue et al: a standard curve for each DENV serotype was developed using four DENV representative strains (one for each serotype). The starting concentration followed a ten-fold dilution of each serotype: serotype 1 (16007) = 2.3×10^6^ PFU/ml; serotype 2 (16681) = 2.5×10^7^ PFU/ml; serotype 3 (IQD1728) = 4.8×10^5^ PFU/ml and serotype 4 (1036) = 4.6×10^5^ PFU/ml.

### Gene expression profile analysis using Affymetrix gene chips

Extraction of cellular RNA, assessment of the integrity and quantity of the extracted RNA, and subsequent processing of the RNA for gene array were performed using the Agilent 2100 Bioanalyzer system (Agilent Technologies, Santa Clara, CA) as previously described [20], [21]. Two chip types were used in the study: Affymetrix HG-U133 plus 2 and HG-Focus. The HG-U133 plus 2 contained 54,675 probe sets, where each set consisted of 11 25-mer probes. The HG-Focus gene chip is consisted of 8,793 probe sets to assess 8,500 transcripts encoding 8,400 full length and fully annotated genes [20], [21]. The 8793 probe sets of the HG-Focus are a subset of the probe sets from HG-U133plus2.

### GeneChip quality control and data normalization

Gene chips with a scaling factor >50 on the dChip software (2005 version) were eliminated from further analysis [21], [22]. Gene chips were also examined using the QA/QC functions built into the Partek software according to the Company's User Manual. Affymetrix CEL files were imported into Partek Genomics Suite Version 6.6 (Partek). The gene chips that failed according to the QC metrics and appeared as outliers using principal component analysis (PCA) were eliminated for further analysis. The CEL files were normalized at the probe level using the Robust Multi-chip Average (RMA) method built into the Partek Genomic Suite software. RMA was used to normalize the microarray data, which leveraged the gene expression assessments made on Affymetrix gene chips. For each probe set, the technique generated an estimated value for the probe and chip effects, which resulted in an overall pattern of probe set values observed in the data set. RMA consisted of 3 calculation applications that address background correction, normalization of the quantiles, and median polish summarization. The gene expression data were expressed as log2 values. The data sets from the two gene chip platforms were normalized and analyzed independently.

### Differentially expressed genes

Differentially expressed genes were analyzed using analysis of variance (ANOVA) using the Partek software. Multi-way ANOVA was chosen based on the number of factors contributing to data variation. The following factors were taken into consideration as variation factors: subject #, scan date (date that the chip image was taken), infecting serotype, illness day (days of illness when the sample was taken), and disease severity (samples associated with the DF or DHF category). Genes that were considered significant only by chance were defined by the false discovery rate (FDR≥5%) and were excluded from further analysis. In this study, the genes with a fold change of >2 or <−2 and a p-value plus a FDR <5% were considered significant. A heat map and PCA were used to visualize the most informative trends by showing the predominant gradients in the data set.

### Gene ontology and functional pathway

Analysis of the gene biological functions and pathways were performed using the pathway analysis modules in the Partek Genomic Suite 6. Two analysis modules were used: gene ontology (GO) [23] and KEGG pathway [24]. The gene ontology/biological process level-5 (GOTERM_BP_5) [25] was used for the GO analysis. The Database for Annotation, Visualization and Integrated Discovery (DAVID), an online program for microarray data mining developed by NIAID was used as a second tool for gene function and pathway analysis.

### Classification

Class prediction was performed using the Partek Model Selection tool in the Partek software. Using this software, nested cross-validations were used to select an optimal classifier and to estimate the accuracy of the optimal classifier when multiple classifiers were considered for a single problem. For the 2-level cross-validation, an “inner” cross-validation was performed to select predictor variables and optimal model parameters, and an “outer” cross-validation was used to produce overall accuracy estimates for the classifier. Following the 2-level cross-validation method, the 1-Level Cross-Validation was used to evaluate multiple models and to select the best model to deploy. In this study, the 2-level nested cross validation process used ANOVA or Shrinking Centroid to filter the data. Multiple groups of variables with specified sizes were evaluated. The best classification model was determined by running the following classifiers: K-nearest neighbors with 1–25 neighbors, Nearest Centroid, Discriminant Analysis, and Support Vector Machine.

## Results

### Clinical information of the study population

From 2006 to 2010, approximately 300 febrile individuals in Maracay, Venezuela, who presented themselves at participating clinics and hospitals or were identified by community-based monitoring, with signs and symptoms consistent with dengue disease, met the study enrollment criteria. These individuals were subsequently enrolled into the study. Approximately 130 subjects were confirmed by PCR of DENV infection and were categorized as DF and DHF patients according to the WHO 1997 dengue disease classification guidelines. DHF patients were recognized based on fever, bleeding, thrombocytopenia (platelet counts ≤100,000) and signs of plasma leakage. Subjects who had ≥3 serially collected samples, 1 at each of the febrile, defervescent and convalescent stages were selected for the study, thus we had 51 DF and 13 DHF subjects providing a sum of more than 200 samples for the study. The demographics of the study participants and their clinical, immunological and hematological characteristics are summarized in Table 1. The timing of the collection, serotype, and severity, related to the samples are presented in Table 2. To assess the statistical difference between DF and DHF, hypothesis testing of 2 independent samples with unknown variances was performed for mean values, and hypothesis testing for 2 sample proportions was performed for percentages. As shown in Table 1, most of the DHF cases met all 4 classification criteria. The percentages of lymphocytes and neutrophils in the peripheral blood of DF and DHF patients were plotted in Figure S1.

**Table 1:** Clinical, immunological and virological information of study cohorts.

Gender and Age	DF (n = 51)	DHF (n = 13)	p value
Sex: Female, No (%)	27 (52.9)	10 (76.9)	0.19
Age: Mean years ±SD (range)	15.3±7.1 (5–32)	19±13.4 (5–49)	0.35
**Infecting Serotype** 1			
DENV-1, No (%)	26 (50.9)	2 (15.4)	0.33
DENV-2, No (%)	8 (15.7)	8 (61.5)	0.06
DENV-3, No (%)	9 (17.7)	2 (15.4)	0.94
DENV-4, No (%)	7 (13.7)	1 (7.7)	0.87
**Pre-existing DENV infection** 2			
None, No (%)	23 (45.1)	2 (15.4)	0.42
One serotype, No (%)	13 (25.5)	1 (7.7)	0.69
Two serotype, No (%)	10 (19.6)	6 (46.1)	0.26
Three serotype, No (%)	4 (7.8)	4 (30.8)	0.41
**Symptoms**			
Hospitalization, No (%)	8 (15.7)	10 (76.9)	<0.01
Temp Max °C ± ST DEV	38.9±0.7	39.1±0.7	0.37
Fever Days ± ST DEV	4.3±1.1	5.2±1.0	<0.01
Headache, No (%)	50 (98.0)	13 (100)	0.61
Shivering, No (%)	43 (84.3)	10 (76.9)	0.58
Rash, No (%)	37 (72.6)	8 (61.5)	0.53
Cough, No (%)	12 (23.5)	4 (30.8)	0.77
Diarrhea, No (%)	10 (19.6)	6 (46.2)	0.26
Nausea, No (%)	19 (37.3)	7 (53.8)	0.45
**Hematology**			
Thrombocytopenia			
(platelet ≤100,000/mm^3^), No (%)	8 (15.7)	12 (92.3)	<0.01
Plasma leakage^3^, No (%)	6 (11.8)	7 (61.5)	<0.01
Bleeding^4^, No (%)	23 (45.1)	13 (100)	<0.01
**Viral Load (Mean ± ST DEV)**			
Day 0 (No)	-	-	-
Day 1 (No)	1.4E+04±2.8E+04 (7)	-	-
Day 2 (No)	7.4E+03±2.8E+04 (27)	3.2E+03±5.4E+03 (3)	>0.05
Day 3 (No)	4.2E+02±1.4E+03 (21)	1.6E+06±4.6E+06 (9)	>0.05
Day 4 (No)	0.7E+01±2.4E+01 (14)	1.1E+04±2.7E+04 (6)	>0.05

1 Serotype for 1 DF subject was missing due to a suspected non-DENV infection.

2 PRNT for 1 DF subject was not done.

3 Elevated hematocrit comparing the highest to the lowest values recorded, or other signs of plasma leakage (pleural effusion, ascites, or other edema).

4 Tourniquet test or signs of epistaxis, ecchymosis, gum bleeding, hematemesis, hemoptysis, genital bleeding, or other bleeding.

**Table 2:** Sample information on two gene chip platforms.*

Array			by	severity		by	serotype	
Platform	G	All	DF	DHF	DENV1	DENV2	DENV3	DENV4
	G0	2	2	0	0	1	1	0
**HG-focus**	G1	4	4	0	2	0	1	1
	G2	21	20	1	14	2	3	2
	G3	19	19	0	9	4	4	1
	G4	24	21	3	12	4	5	4
	G5	23	22	1	14	1	3	3
	G6	26	25	1	18	0	7	1
	G7	50	47	3	25	6	9	6
	**Total**	**166**	**157**	**9**	**95**	**18**	**33**	**18**
	G0	0	0	0	0	0	0	0
**HG-U133**	G1	2	2	0	0	2	0	0
**plus 2** **	G2	8	5	3	1	5	1	1
	G3	14	7	7	2	8	1	2
	G4	18	8	10	3	10	3	2
	G5	9	4	5	4	5	0	0
	G6	22	11	11	7	7	5	3
	G7	24	12	12	7	11	3	3
	**Total**	**97**	**49**	**48**	**24**	**48**	**13**	**11**

* Tables shows number of samples per category.

** Among 97 samples tested on HG-U133plus2, 50 were also tested on HF-focus.

### Day-to-day host gene expression depicted two sub-phases during the process of the disease

A total of 166 specimens from 47 DF and 3 DHF subjects were used to study the dynamics of host responses using the HG-focus gene chip platform (Table 2).

**Figure 1:**
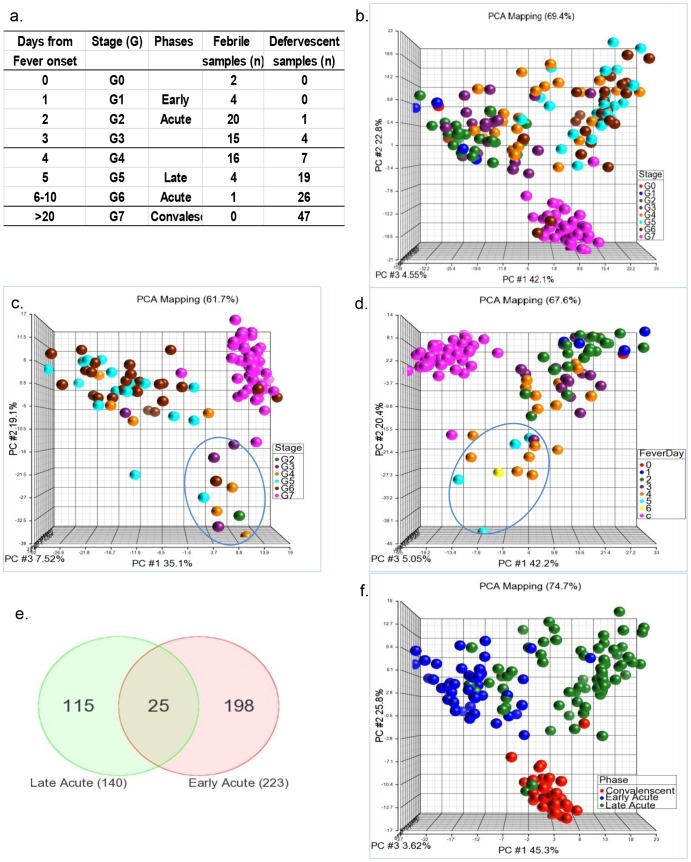
Daily gene expression patterns define 2 subtle phases during the disease process. a) Illness days with corresponding stages (G) and phases for samples on the HG-focus platform. The number of febrile and defervescent samples in each G was shown. b) A global overview of the gene expression pattern in G0 to G7. Using convalescent samples (G7) as a baseline, significant genes in each stage (from G0 to G6) were detected using multi-way ANOVA. PCA was performed using all of the significant genes found in G0 to G7 on the HG-focus platform. All of the samples (represented by dots) were included in the PCA. c) PCA was performed on the defervescent samples only. d) PCA was performed on the febrile samples only and are indicated according to the number of fever days. e) Samples were reassigned to the early acute, late acute and convalescent phases. Differentially expressed genes in the early acute *vs.* convalescent or late acute *vs.* convalescent phase were detected using multi-way ANOVA. Shared genes between the early and late acute phases were represented by a Venn diagram. f) PCA was performed using 313 phase-specific genes.

To analyze the dynamics of the host response in DENV-infected individuals, samples obtained at different illness days were grouped together into stages (G). Group sizes (# of specimens) were shown by G and also by serotype or severity (Table 2). As illustrated in Figure 1a, G0 was the day of fever onset, G1–G5 corresponded to 1–5 days from fever onset, G6 was day 6–10, and G7 was the convalescent time point day ≥20. As shown in Figure 1a, each G had ≥19 samples except G0 and G1. Using levels of gene expression at G7 as the baseline, significantly up- or downregulated genes were detected at each G using 5-way ANOVA (Subject #, scan date, severity, stage, and serotype as 5 factors). The number of significant genes in each G from G0–G6 was 360 (G0), 320 (G1), 92 (G2), 136 (G3), 122 (G4), 198 (G5), and 152 (G6), respectively. Combined, these genes consisted of a total of 615 genes (7.0% of 8793 probe sets on the FG-focus platform) representing the total up- or down regulated genes activated throughout the entire illness period. Visualizing the expression patterns of the 615 in all of the samples at all time points using PCA, we observed that gene expression patterns gradually shifted by stages from G0 to G6 (Figure 1b). Within this gradual shift, G0, G1, G2, and G3 formed a cluster that was clearly distinguishable from that of G5 and G6. The samples from G4 bridged between the two clusters.

To determine whether the change of gene expression, especially the two distinguishable clusters, was modulated by the presence or absence of fever, we performed PCA on only the defervescent samples. As shown in Figure 1c, the majority of the G4–G6 defervescent samples clustered together, while 4/5 G2–3 and some G4 defervescent samples separated (Figure 1c), suggesting that defervescent samples from a shorter febrile duration (2–3 days) maintained gene expression patterns that were more similar to those of early febrile samples. Similarly, when PCA analysis was performed only on the febrile samples (Figure 1d), it was clear that all of the 5 G5–6 samples, together with a few of the G4 febrile samples separated from the rest of G0–G4 febrile samples, and maintained a pattern more similar to that of late defervescent samples.

Based on the 2 distinguishable clusters shown in Figure 1b, we regrouped the samples into early acute (G0–G3), late acute (G4–G6) and convalescent phases (G7). A second 4-way ANOVA was performed by comparing the gene expression in the early acute *vs.* convalescent and late acute *vs.* convalescent phases. We identified 223 and 140 significant genes that were differentially expressed in the early acute and late acute phase, respectively (Figure 1e, Table S1 and S4). Only 25 genes (∼8%), as shown by a Venn Diagram (Figure 1e), were shared between the early and late phase, indicating persistent expression of these genes; whereas the majority (∼92%) of the genes (198+115 = 313) were uniquely expressed in either the early or late phase (gene list shown in Table S7). These phase-unique markers showed a better separation of the 3 phases (Figure 1f): early acute, late acute and convalescent.

To validate our observations, we performed a similar analysis for the samples assayed using another chip type: HG-U133plus2 (sample information shown in Table 2), and similar results were obtained (Figure S2a–c). We further separated the DF and DHF samples and analyzed them separately. The gradual change in gene expression over time and the clustering of G1–G3 and G4–G6 were both observed in the DF and DHF groups (Figure S3), suggesting that the time of sampling contributed most significantly to gene expression variation.

### Functionally categorizing differentially expressed genes in early and late acute phases

To understand the functions of the differentially expressed genes identified by ANOVA, gene ontology (GO) and Kegg pathway analysis was used to annotate the functional profiles. The entire list of GO categories in the early and late acute phases with a pathway p-value <0.01 and with more than 3 involved genes are shown in Tables S2, S3, S5, and S6. Listed in Table 3 are the most significant pathways with the highest enrichment scores as detected using the HG-focus gene chip platform. As shown in Table 3, genes upregulated in G0–G3 were related to the innate immune pathways, type-I interferon-mediated signaling, cytokine-mediated signaling, response to virus, chemotaxis, and inflammatory responses. Gene down-regulated in G0–G3 were related to gene transcription and translation, cellular protein metabolic processes, structural constituent of ribosome, viral transcription, and viral infectious cycle. Genes upregulated in G4–G6 were related to mitotic cell cycle, cell division, mitosis, DNA replication, chromosome, spindle organization, phosphatidylinositol-mediated signaling. Strikingly, there was minor overlap between early acute and late acute phases in terms of gene functionality.

**Table 3:** Common and differential GO pathways in early acute and late acute phases.

Phase	Up-regulated GO pathways	Down-regulated GO pathways
**Early Acute**	type I interferon-mediated signaling pathway	translational elongation
	cytokine-mediated signaling pathway	translation
	immune response	structural constituent of ribosome
	interferon-gamma-mediated signaling pathway	viral transcription
	innate immune response	translational termination
	double-stranded RNA binding	cellular protein metabolic process
	response to virus	viral infectious cycle
	chemotaxis	gene expression
	inflammatory response	endocrine pancreas development
	defense response to virus	ribosome
**Late Acute**	mitotic cell cycle	positive regulation of nitric oxide biosynthetic process
	cell division	positive regulation of fever generation
	mitosis	peroxidase activity
	spindle organization	haptoglobin-hemoglobin complex
	cell cycle	sequestering of triglyceride
	DNA replication	positive regulation of calcidiol 1-monooxygenase activity
	M phase of mitotic cell cycle	humoral immune response
	phosphatidylinositol-mediated signaling	oxygen transport
	chromosome	regulation of I-kappaB kinase/NF-kappaB cascade
	cell cycle checkpoint	
**Both phases**	cytokine-mediated signaling pathway	
	type I interferon-mediated signaling pathway	
	response to virus	

The GO analysis using genes expressed on HG-U133plus2 showed same GO pathways as those on HG-focus, reemphasizing the reproducibility of the data (Table S8).

Furthermore, two of the top Kegg pathways representing the early acute and late acute phases were the Systemic Lupus Erythematosus (SLE) (Figure S4) and Cell Cycle (not shown) pathways, respectively. SLE is an autoimmune disease associated with type III hypersensitivity. It is triggered by the precipitation of antibody immune complexes to cells and tissues, causing complement activation, immune cell activation, and inflammation, and results in tissue and organ damage. Genes encoding complement components C2, C1q and C1r and genes associated with antigen processing and presentation, cytokine-cytokine signaling, T cell receptor signaling, cell adhesion, complement coagulation, and all those marked with a red star, were present in G0–G3.

### Genomic signatures predictive of 3 dengue disease phases with 91% accuracy

**Figure 2:**
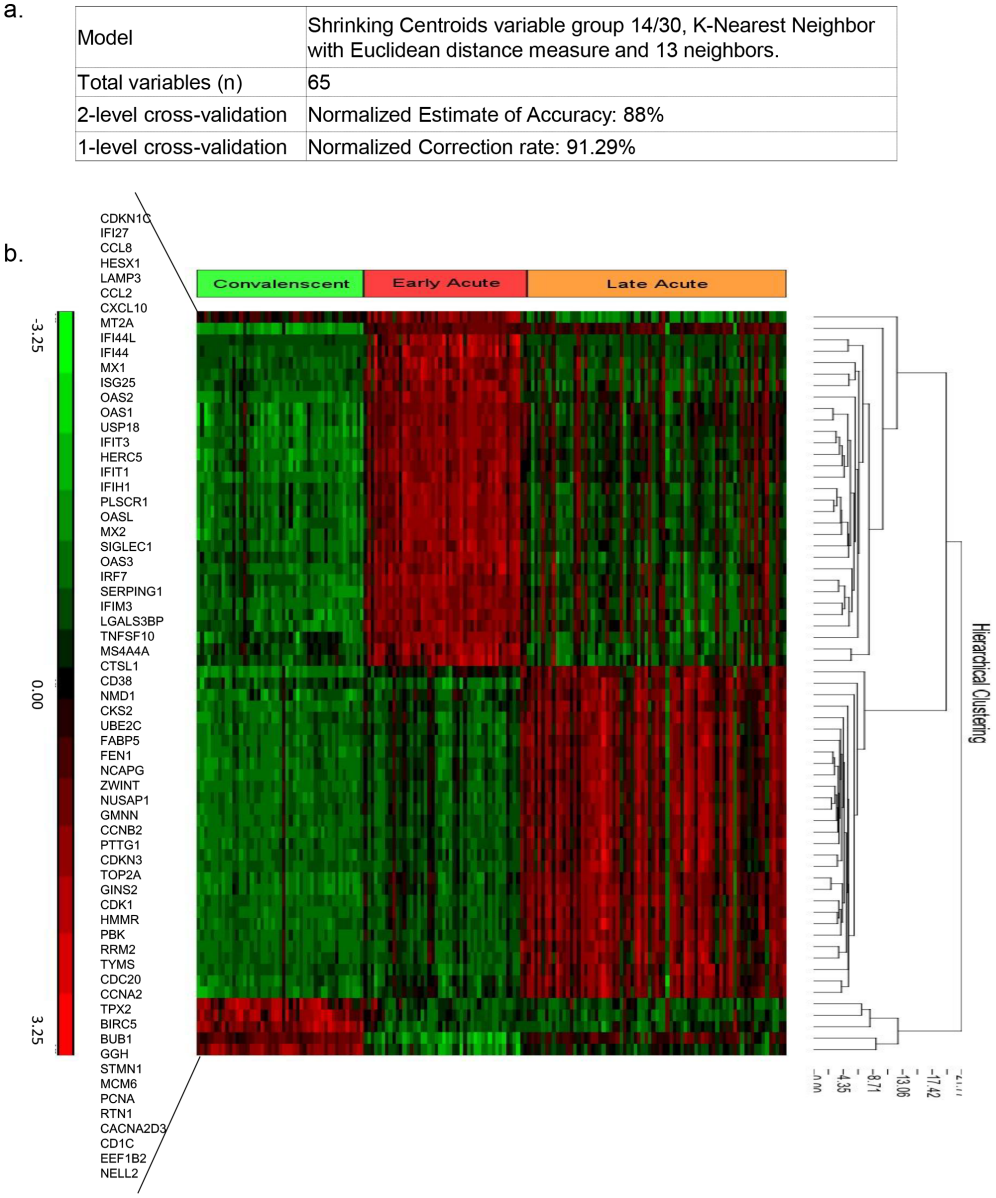
2-level and 1-level cross-validation identified a top classification model and 65 gene signatures for the prediction of 3 phases. a) Information of the model selection showing 65 variables and a top classification model with 2-level and 1-level normalized correction rates. b) Heatmap of the 65 signatures.

For the classification of dengue disease phases, 2-level cross-validation using all of the data on the HG focus chips returned a Normalized Accuracy Estimate = 88%. The 1-level cross-validation returned a top model which used 65 variables and yielded a Normalized Correction Rate of 91% (Figure 2a). These results showed that a set of 65 variables classified early acute, late acute and convalescent phases with an accuracy of 91% (Figure 2b). Among these 65 genes, 23 and 27 variables were unique gene signatures for the early acute and late acute phase, respectively, whereas 15 variables were expressed in both the early and late acute phases.

We also performed classification analysis with samples on the HG-U133plus2 platform and found that 55 out of these 65 phase classifiers were also (Table S9) found on this platform.

### Sequential waves of marker expression

**Figure 3:**
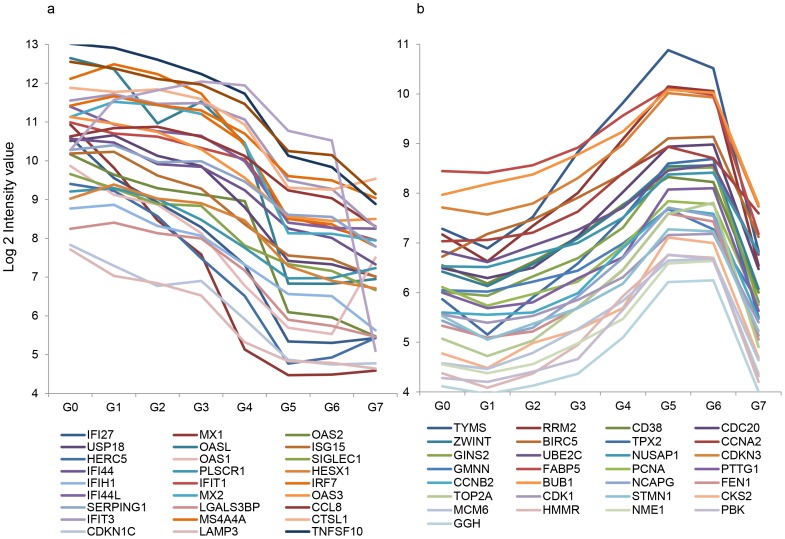
Two waves of gene expression over the disease stages. Median marker expression intensity (log 2 value) of the genes detected in the early and late acute phases from G0 to G7 was shown.

The dynamics of the expression of these 65 markers from G0–G7 were analyzed and shown in Figure 3. Markers belonging to the early acute phase peaked at G0–G1 and gradually declined to baseline around G4–G5; whereas markers unique for the late acute phase emerged at G4 and peaked at G5–G6. These results showed two sequential waves of genes representing two host response periods: an innate response period followed by a cell mitotic cycle period. The cross-point of the two waves was at G4.

### A list of the top 7 genes with 96% accuracy in predicting DF vs. DHF

We further analyzed the data on the HG-U133plus2 chips for gene expression related to DF *vs.* DHF. Classification analysis was first performed using all of the genes on the HG-U133plus2 chip for samples in G0–G3. A total of 140 genes (Table S10) classified DF *vs.* DHF with a correction rate of 86% using the classification methods of K-nearest Neighbor with Euclidean distance and 3 neighbors. The fold changes of the 140 genes between DF and DHF were >2 or <2 with p (FDR) <0.05 by ANOVA analysis (Table S10). Among these 140 genes, 79 genes (59%) showed higher levels of expression in the DF samples compared to the DHF samples; whereas 61 genes (41%) showed higher levels in the DHF samples compared to the DF samples (Table S10). Antigen processing and presentation of the peptide or polysaccharide antigen via MHC class II, MHC class II protein complex, interferon-gamma-mediated signaling pathway, T cell receptor signaling pathway, MHC class II receptor activity, and T cell co-stimulation were among the top functional bio-pathways with a higher level expression in DF specimens compared to DHF specimens (Table 4).

**Table 4:** GO pathways of gene signatures which differentiated DHF from DF.

Pathways	Up/down	Enrichment	Enrichment
	Df vs DHF	Score	P value
antigen processing and presentation of peptide or polysaccharide antigen via MHC class II	Up	24.2904	0.000000
MHC class II protein complex	Up	23.8583	0.000000
interferon-gamma-mediated signaling pathway	Up	21.9241	0.000000
T cell receptor signaling pathway	Up	17.1104	0.000000
MHC class II receptor activity	Up	16.7737	0.000000
cytokine-mediated signaling pathway	Up	15.0755	0.000000
lysosomal membrane	Up	14.6264	0.000000
T cell costimulation	Up	14.5901	0.000000
Golgi apparatus	Up	11.4283	0.000011
type 2 fibroblast growth factor receptor binding	Down	10.1483	0.000039
regulation of branching involved in salivary gland morphogenesis by mesenchymal-epithelial signaling	Down	10.1483	0.000039
surfactant homeostasis	Down	9.38874	0.000084
branching involved in salivary gland morphogenesis	Down	9.00746	0.000122
killing of cells of other organism	Down	9.00746	0.000122
defense response to fungus	Down	8.84125	0.000145
phosphatidylcholine biosynthetic process	Down	8.68795	0.000169
defense response to bacterium	Down	8.68049	0.000170
hair follicle morphogenesis	Down	7.50439	0.000551
response to virus	Down	6.96488	0.000944

**Figure 4:**
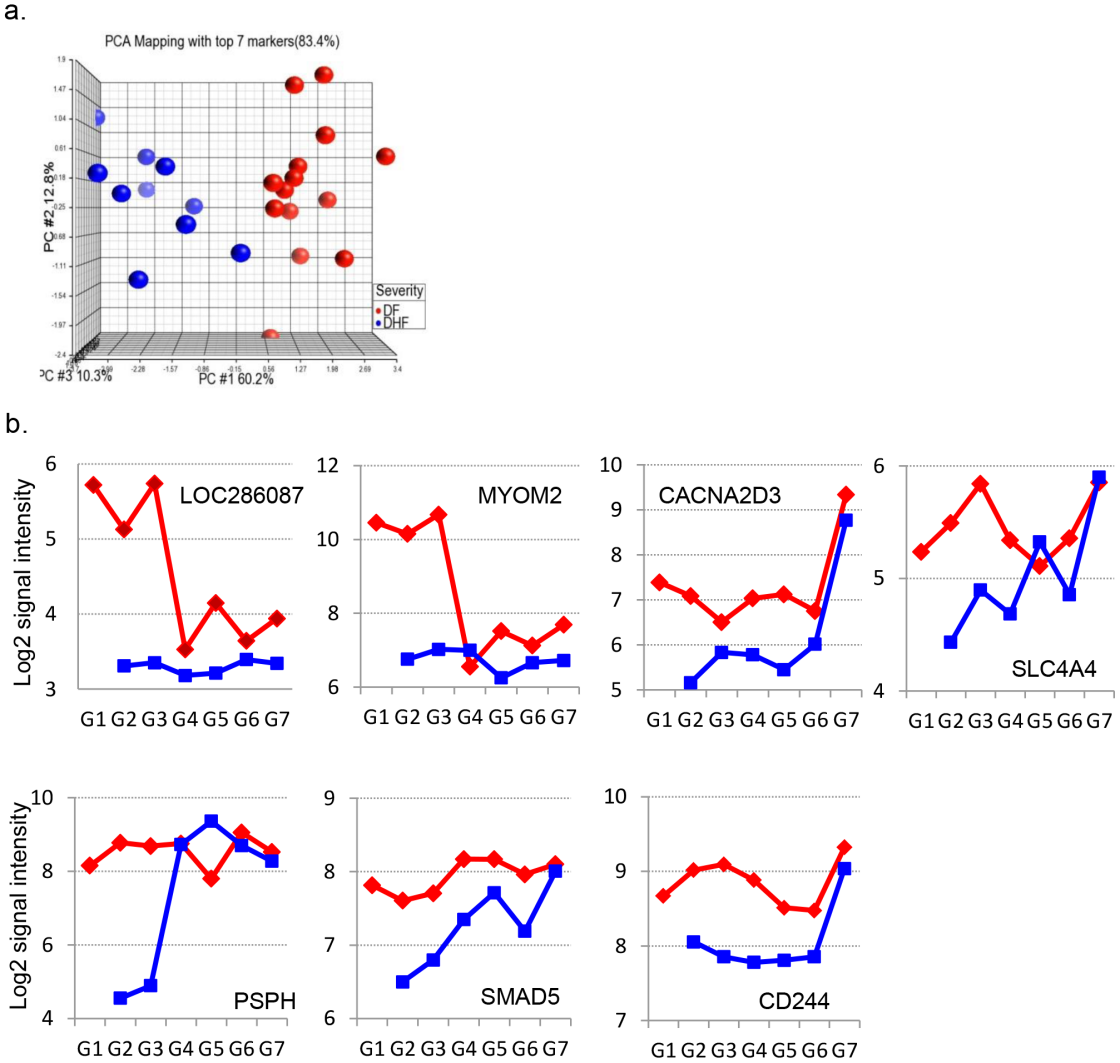
Classification of DF and DHF. a) The expression pattern of the top 7 genes (selected from 140 genes) is indicated using PCA. b) The dynamic expression (shown by median Log2 gene expression intensity) of 7 DF-DHF signatures from G1 to G7.

Since our ultimate goal was to discover markers for the early diagnosis of DHF, we sought markers expressed differently between DF and DHF throughout G1 to G3, but not the ones different in only one or two stages. The median level of gene expression for each of the 140 genes in G1, G2 and G3 for the DF and DHF group, respectively, was analyzed. There were 56 (out of 140) genes met this criterion with a fold change of >2 or <−2 throughout G1 to G3 compared to G7. Ranking of the 56 genes revealed 7 genes (Table 5) that differentiated DF from DHF with a 96% accuracy. PCA using these 7 genes clearly segregated the DF from the DHF samples (Figure 4a). The dynamic expression of these 7 genes was shown in Figure 4b.

**Table 5:** List of top 7 genes differentiating DF from DHF with 96% accuracy.

Probeset	Gene	Gene Title	p-value	Fold-Change	Up/down
ID	Symbol		(DF vs. DHF)	(DF vs. DHF)	
1556842_at	LOC286087	hypothetical protein LOC286087	0.0002	4.0401	DF up vs DHF
203908_at	SLC4A4	solute carrier family 4, sodium bicarbonate cotransporter, member 4	0.0001	2.0548	DF up vs DHF
205048_s_at	PSPH	phosphoserine phosphatase	0.0000	8.1003	DF up vs DHF
205826_at	MYOM2	myomesin (M-protein) 2, 165 kDa	0.0004	4.9076	DF up vs DHF
219714_s_at	CACNA2D3	calcium channel, voltage-dependent, alpha 2/delta subunit 3	0.0008	2.4949	DF up vs DHF
220307_at	CD244	CD244 molecule, natural killer cell receptor 2B4	0.0001	2.1211	DF up vs DHF
225219_at	SMAD5	SMAD family member 5	0.0003	2.1587	DF up vs DHF

## Discussion

The samples collected in this study spanned from as early as the first day of fever onset to early defervescent to the convalescent period. They provided an exceptional opportunity to investigate the whole spectrum of host responses during the disease process. The samples from earliest time points were particularly valuable in understanding the differences in pathology between DF and DHF at early stages of disease.

### Clinical, immunological and hematological information of DF and DHF cohorts

The classification of DF and DHF cases played a key role in this study. We followed the 1997 WHO guidelines: persistent high fever, hemorrhagic manifestations (spontaneous bleeding), thrombocytopenia (platelet counts ≤100000/mm^3^), and signs of plasma leakage (≥20% increase in hemoconcentration, pleural effusion or ascites). Most of our DHF cases met all 4 criteria.

Other differences characterizing DHF from DF included a prolonged fever duration (5.2 *vs.* 4.3 days), higher hospitalization rate, higher secondary infection rate, and a trend of higher viremia titers, which were all consistent with our current knowledge [9], [26], [27].

In all patients, there was a decrease in lymphocytes but an increase in neutrophils within the first 1–3 days of illness compared to the convalescent baseline (G7). Changes in the lymphocytes and neutrophils were significantly more pronounced in the DHF compared to the DF cases. Potts et al attempted to use hematological measures for diagnosis of severe dengue illness [28]. They found that higher counts of neutrophils and lower counts of white blood cells within 3 days from the onset of illness predisposed people at a higher risk of DHF. Mechanistically, early stage T cell apoptosis [29], [30] demonstrated by Green et al. may account for the lower lymphocyte counts. T cell apoptosis may also account for the lack of functional CD8^+^ T cell cytokine production [31], and lack of T cell proliferation at the febrile period of dengue illness [32]. Taken together, these data support the role of cellular immunity in the defense against dengue disease severity, and provided some biological insights on our gene array results, which are later discussed. Neutrophils are one of the first cells to migrate to infection sites; they play an important role in the control of various bacterial and viral infections though phagocytosis and cytokine/chemokine production [33]. Neutrophils release an array of cytokines and chemokines to impact the functions of other cells of the innate as well as the acquired immune system [34], [35]. The changes of neutrophil counts in early stages may provide mechanistic insights for our gene array study, as the chemokine and inflammatory cytokine responses are the top functional gene pathways in early acute phase.

### Two waves of gene expression representing two distinctive phases of disease process

We observed a gradual evolution in the gene expression patterns over time from G0 to G6 with a more significant change at G4, resulting in the separation of the early acute (G0–G3) and late acute phases (G4–G6). This was independent of disease severity and was incompletely associated with fever status. The early acute and late acute phases were represented by two waves of functionally distinct gene clusters: the innate immunity followed by cell cycle. Strikingly, only 10% genes were shared between the two phases, and >90% of the genes were unique for either the early or late phase.

A number of published dengue gene array studies [11], [12] have highlighted the importance of sampling time and addressed the difference with respect to timing. However, there is not a uniformed and clear method in the timing of the samples. Some studies used ≤72 hours to define the early phase, while others treated samples from various days as one group. Furthermore, due to the limitation of their sample bank (most of the studies had samples from day 3–6), the studies did not show an evolving picture of gene expression on a day-to-day basis, and were mostly unable to capture both waves of gene expression [11], [13], [36], [37]. Their findings highlighted a specified period of dengue disease. To the best of our knowledge, we are the first study to present the host response to dengue infection as an entirety.

Nevertheless, when we examined our results and those of others by breaking down to separate phases, we found that our results and those of others were mutually supportive. Hoang et al. had used samples collected within 72 hours of the illness from Vietnam, and had identified major functional pathways, response to virus, immune response, innate immune response and inflammatory responses, which were nearly identical to those found in our study. Fink et al. conducted a study on dengue febrile subjects in Singapore [11]. Approximately 50% of their 32 reported innate immunity genes, including TNF, IP-10 (CXCL10), I-TAC (CXCL11), Stat 1, OAS1, OAS2, OAS3, OASL, IFIH1, IFI44, UBE2L6, UPS18, HERC5, ISG15, PSMB9, MxA (Mx1) were also identified during the same period of disease G0–G3 in our study. More interestingly, IP-10 (CXCL10) gene was one of the most upregulated genes demonstrated in both ours and Fink's study. In addition, Simmons et al. used samples from day 3–6 with a mean length of illness of 4.6 days from hospitalized patients in Ho Chi Minh City, Vietnam [12]; Loke et al. studied gene expression using samples within 3–6 days of illness from children in Nicaragua. The presence of genes related to cell cycle, protein metabolism and nucleic acid metabolism in these two studies confirmed our observation of the late acute phase. Our results suggested that future studies should consider carefully the time of sample collection since host responses at early acute and late acute phases showed little in common.

### The two waves of genes possibly implicate two stages of host response: anti-viral response and post-infection recovery

The switch in major biological processes from innate immunity to cell mitotic cycle at G4 occurred at G4. It appeared that this switch coincided with a decrease in viremia. As shown in Table 1, viremia decreased to low levels in most dengue cases at around G3–G4. Genes related to the viral replication cycle, RNA synthesis and RNA transcription were downregulated in G0–G3, which coincided with the upregulation of innate immune responses during this phase. It is known that anti-viral inflammatory cytokines and soluble factors, IL-1, -6, -8 and -10 or TNFα and γ, MIP-1α and γ or VEGF [38]–[42], and the nitrogen and oxygen species [43], were present in patient sera. Our study suggested a vital role of type I IFN responses, anti-viral responses, as well as chemokines, chemotaxis and complement activation in suppressing viral replication.

Apoptosis of DENV-infected cells has been demonstrated *in vitro* or *in vivo* in almost every single type of cells infected by DENV. Studies using PBMCs from dengue patients show that T cells undergo activation and apoptosis during the acute illness phase when viremia was present. Impaired CD8+ T cell activity [31], decreased circulation of CD4^+^ and CD8^+^ T cell counts, impaired T cell proliferation [30], [32], and increased T cell apoptosis, were all demonstrated during the acute phase. Restoration of T cell activities, T cell counts, and cytokine production was detected from day 5 onward [29]–[32], which coincided with the absence of viremia *in vivo*. In our study, the patients also showed a decrease in lymphocytes during G0–G3, which subsequently returned to above baseline levels. Although they were not the top categories, apoptotic pathways, including the activation of pro-apoptotic gene products, response to unfolded proteins, activation of caspase activity, release of cytochrome C from the mitochondria, and induction of apoptosis by extracellular signals were upregulated during the early acute phase, supporting previous *in vitro* and *in vivo* observations on cell apoptosis during early days of infection. The subsequent upregulation of genes related to DNA synthesis and the mitotic cell cycle in G4–G6 suggests a period of immune cell recovery, which may begin when viremia has significantly decreased.

### Biological pathways identified using proteomics and genomic pathways

Our previous proteomics study revealed several serum biomarkers that predicted DHF. One of these markers was the complement component C4a [44]. Although direct expression of C4a gene was not detected, its upstream complement components, C1q and C2, and other factors such as HF1, BF1, CD59 and SERPING1 were detected in the early acute phase. The complement system can be activated by a classical immune complex (IC) -dependent pathway, an alternative pathway and a lectin pathway. Activation of the complement system restricts viral or bacterial infection, but it also promotes strong inflammatory responses. Complement C1q- or C3 have been shown to eliminate ADE [45]. In dengue patients, the peak presence of C3a and C5a coincided with the onset of shock and leakage. In addition, the levels of C3a correlated well with disease severity [46]. By capturing genes in the complement pathways, our study highlighted the involvement of the complement system in the early acute phase. Since most DHF cases are secondary infectious cases, the involvement of the complement system in dengue disease severity requires further investigation.

### Markers of disease severity

We found a set of 140 genes that distinguished DF from DHF with 86% accuracy. Genes expressed more abundantly in DF were associated with antigen processing and presentation, such as the MHC class II protein complex, interferon-gamma-mediated signaling, T cell receptor signaling, and T cell co-stimulation pathways.

Seeking gene markers for severe dengue disease has been an exclusive goal in nearly every gene array study conducted. For those studies which used samples collected from day 3–6, their results differed from our findings [13]. Popper et al. recently performed a second gene array study in Nicaragua, where they investigated gene expression in a day-to-day manner [37]. They showed that on fever day 3, lower levels of IFN-stimulated gene transcripts were associated with the development of DSS. The results from this study showed some consistency with our findings. Nascimento et al. conducted a gene array study on a well-characterized dengue cohort from Recife, Brazil [47]. Their results also showed that at early stages of DENV infection, the genes involved in the effector functions of innate immunity were weaker in DHF patients. Furthermore, Devignot et al. showed that genes related to T and NK cell responses were decreased and genes related to anti-inflammatory and repair/remodeling were increased in DSS patients in a study in Cambodia [48]. Overall, the results generated from our study and from those previously reported illustrate an association of IFN-γ and T cell immunity with lower risk of DHF.

In our study, the majority (61.5%) of DHF cases were caused by DENV-2. In contrast, 50% DF cases was due to infection with DENV-1. We were unable to identify differences in the gene expression pattern between any of the two serotypes (data not shown). Thus, different serotypes may not be the main cause underlying the differential gene expression patterns associated with DF or DHF.

The association of cellular immunity with DF, but not DHF, strengthened the protective role of cellular immunity against the severity of dengue disease. Since most of the DHF cases were secondary infections, theoretically, cellular immunity should be more rigorous due to the presence of T cell memory. It is known that DENV primarily infects monocytes, macrophages and dendritic cells. These cells are antigen-presenting cells responsible for antigen processing and presentation. Apoptosis caused by DENV infection of these cells may account for the decrease in lymphocytes in the peripheral blood during the early acute phase of the disease and may explain the weakened gene expression of cellular immunity in DHF patients.

For future diagnostic purposes, we selected 7 genes that were able to differentiate between severe and non-severe dengue disease with a high accuracy (96%). These genes were selected on the basis of their consistent expression throughout the early acute days (G1 to G3). The CD244 gene encodes a cell surface receptor expressed on natural killer (NK) cells (and some T cells) that mediate non-major histocompatibility complex (MHC) restricted killing. The interaction between NK-cells and target cells via this receptor was thought to modulate NK-cell cytolytic activity. The SMAD5 gene encoded protein is involved in TGF-β signaling, which results in an inhibition of the proliferation of hematopoietic progenitor cells. In addition, CD244 and SMAD5 genes were both downregulated in DHF subjects. The CACNA2D3 gene encodes a member of the alpha-2/delta subunit family, a protein involved in the voltage-dependent calcium channel complex.

### Conclusion

To the best of our knowledge, this study was the first study to present a systemic analysis of the full dynamics of the host response in dengue clinical subjects. This study was completed using two chip platforms and obtained highly consistent results between the two platforms. The gene microarray analysis was supported by clinical observations, comprehensive hematological test results, as well as viremia and immunology data. This study also provided solid data to highlight the importance of the timing in the collection of the clinical samples. This study also strengthened the role of IFN-γ and T cell immunity in the defense against DHF. Our results will advance the understanding of the DENV-mediated disease progression, which will provide enormous support for future clinical research, diagnostics and vaccine development.

### Gene Expression Omnibus accessing number

GSE43777

## Supporting Information

Checklist S1 STROBE checklist.
(DOC)

Figure S1 Changes of lymphocytes and neutrophils in groups of DF and DHF patients. Means and standard deviation of lymphocytes and neutrophils for DF group (in blue) and DHF group (in red) were shown. Unpaired student t tests were performed to compare the differences between DF and DHF groups and p value were shown.
(PPTX)

Figure S2 Daily gene expression patterns as detected using HG-U133 plus 2 chips defined 2 subtle phases for dengue acute illness. Using convalescent samples (G7) as a baseline, significant genes in each stage from G0 to G6 were defined as the fold change between Gn to G7: >2 or <−2 and p-value with false discovery rate (FDR) <0.05. S1a). PCA performed with significant genes in all of the samples within each individual G from G0 to G7. S1b) ANOVA analysis revealed overlapping and non-overlapping genes between the early acute and late acute samples on the HG-U133 plus2 platform. S1c) PCA was performed on non-overlapping 1882 phase-specific genes.
(PPTX)

Figure S3 Gene expression patterns were analyzed separately for groups of DF and DHF subjects. Using convalescent samples (G7) as a baseline, significant genes in each time point were detected using multi-way ANOVA for DF or DHF group. PCA was performed using all of the significant genes found in G0 to G7 within each group on the HG-U133 plus 2 platform. a) PCA profiling for the DF group. b) PCA profiling for the DHF group.
(PPTX)

Figure S4 Functional analysis of genes expressed in the early acute phases of the Kegg pathway. Data shown were obtained from the HG-U133plus2 platform. The red stars mark the genes or pathways present in the data.
(PPTX)

Table S1 List of genes significantly changed in the early acute phase as detected using the HG-focus platform.
(PPTX)

Table S2 GO categories upregulated in the acute phase (P<0.05) as detected using the HG-focus platform.
(PPTX)

Table S3 GO categories downregulated in the acute phase (P<0.05) as detected using the HG-focus platform.
(PPTX)

Table S4 List of genes significantly changed in the late acute phase as detected using the HG-focus platform.
(PPTX)

Table S5 GO categories upregulated in the late acute phase (p<0.05) as detected using the HG-focus platform.
(PPTX)

Table S6 GO categories downregulated in the late acute phase (p<0.05) as detected using the HG-focus platform.
(PPTX)

Table S7 Overlapping genes between the early and late acute phases as detected using the HG-focus platform.
(PPTX)

Table S8 Major GO pathways in the early acute and late acute phases as detected using the HG-U133plus2 gene chip.
(PPTX)

Table S9 A set of 65 genes classifying the early and late acute and convalescent phases.
(PPTX)

Table S10 A set of 140 genes differentiating DF vs. DHF as detected using the HG-U133plus2 gene chip.
(PPTX)
